# Economic Burden Imposed on Stroke Patients and Its Determinants: A Cross‐Sectional Study From Semnan, Iran, in 2024

**DOI:** 10.1002/hsr2.73338

**Published:** 2026-10-05

**Authors:** Masood Bagheri, Sayed Amir Mortazavi, Farid Gharibi, Yegane Partovi

**Affiliations:** ^1^ Clinical Research Development Center, Imam Khomeini and Mohammad Kermanshahi and Farabi Hospitals Kermanshah University of Medical Sciences Kermanshah Iran; ^2^ Social Determinants of Health Research Center Semnan University of Medical Sciences Semnan Iran; ^3^ Department of Health Services Management, School of Nursing and Midwifery Semnan University of Medical Sciences Semnan Iran; ^4^ Department of Public Health, School of Health Ardabil University of Medical Sciences Ardabil Iran

**Keywords:** chronic diseases, cost of illness, economic burden, stroke

## Abstract

**Background:**

Stroke is a disease that, due to its nature, can impose various costs on patients. Therefore, identifying the amount and types of patient costs is essential to evaluate the level of social and economic protection provided by relevant institutions.

**Objective:**

This study examined the financial burden imposed on stroke patients and its determinants.

**Methods:**

This descriptive‐analytical study was conducted cross‐sectionally in 2024. The study included 270 participants, selected based on data recorded in healthcare systems and through a census approach. The data collection tool was a standardized questionnaire previously used in similar studies. We first analyzed the collected data descriptively and then analytically. We used *t*‐tests and ANOVA to assess statistical relationships between demographic/background variables and the imposed costs (depending on whether the variables were binary or multi‐level). To evaluate significant differences between defined groups, we applied Tukey's post hoc test.

**Results:**

On average, stroke patients and their households bear an annual cost of 561 million IRR ($1514), and the total economic burden of stroke on patients and their households in Semnan in 2024 is estimated at approximately 151,557,260,000 IRR ($408,755.8). About 60% of these costs are direct medical expenses, while direct non‐medical and indirect costs comprise about 20%. In the analytical section, the study found significant associations between total costs and variables such as marital status, employment status, household income level, having supplementary health insurance, and the patient's experience of stroke complications (*p* < 0.05).

**Conclusions:**

Most stroke patients face a wide and significant range of healthcare costs that they cannot afford due to their sharply reduced ability—and that of their family members—to participate in economically productive activities, along with generally low‐income levels in Iran.

## 1 Introduction

Disease occurrence, in addition to causing significant pain and suffering for the patient and their household, incurs various costs [1]. Therefore, it is a serious responsibility of health systems to ensure that the community is not burdened with high costs due to illness and to provide financial protection for patients who lack the means to pay [2, 3]. Research shows that the chances of facing high healthcare and disease‐related costs are much greater with chronic, complicated illnesses or major surgeries than with other conditions [4, 5]. For example, while about 13% of households in China without a member suffering from non‐communicable diseases face catastrophic healthcare expenses, families with such a member face this burden at a rate of 34% [6]. Moreover, the reduced earning capacity of the patient due to illness and the household's decreased income caused by caregiving responsibilities constitute additional economic consequences called indirect costs, which are viewed as a form of opportunity cost [7, 8].

Stroke is a chronic and disabling disease that, due to its high prevalence and the severe, long‐lasting complications it causes in patients, is regarded as a significant public health issue worldwide [9]. Each year, over 15 million stroke cases occur globally, with one‐third resulting in death and another third leading to permanent disabilities among survivors [10]. Stroke patients utilize healthcare services significantly more, incur higher care costs, and have lower employment rates [11]. Additionally, stroke survivors often need long‐term medical and personal care because of the health complications and disabilities caused by the stroke [12]. Therefore, besides its serious health challenges, stroke also creates widespread economic and social impacts on families, the healthcare system, and society as a whole [13].

In 2017 alone, strokes cost €60 billions across European countries, with 63% of that amount attributed to the four most populous nations—Germany, France, Italy, and the United Kingdom [14]. Given the rising incidence of strokes and the increasing costs due to an aging population and more complex healthcare services, an upward trend in the financial burden of this disease is expected [14]. Consequently, the annual cost to society from stroke in the United States is projected to reach $240.67 billion by 2030 [15]. Therefore, conducting cost‐analysis studies on major diseases like stroke is critically important for gaining accurate insights into the amount and distribution of expenses [16]. Moreover, research indicates that studies addressing indirect costs—particularly those examining the degree and severity of disability caused by stroke—are extremely rare [10, 17].

Recently, our research team studied the incidence of health care costs based on the out‐of‐pocket share of household expenses among stroke patients and found that 67% face health care costs. However, the main point is that out‐of‐pocket costs are not only the costs imposed on patients and their families, but also a range of direct medical, direct non‐medical, and indirect (opportunity costs) costs imposed on patients and their families, the total of which represents the economic burden imposed on patients and their families [18]. Literature review results show that despite the evident importance of this issue, no studies have been done in Iran on the economic burden faced by stroke patients and their households or on identifying vulnerable groups in this area. Therefore, this study aimed to investigate the economic burden on stroke patients and their families and identify its determinants.

## 2 Methods

### 2.1 Setting and Participants

The current descriptive‐analytical study was conducted cross‐sectionally in 2024. We identified 270 participants from data recorded in the care systems of Semnan University of Medical Sciences and included them using a census approach. Inclusion criteria included a confirmed stroke diagnosis and at least 1 year of care. Exclusion criteria comprised having severe related conditions, such as congestive heart failure or chronic kidney failure, at the same time. Of the 308 patients with stroke, 32 were excluded based on the defined inclusion and exclusion criteria. In this study, all eligible stroke patients (276) were included, and with coordination and follow‐up by the research team, 270 responded (response rate 97.8%) (Figure 1.

**Figure 1:**
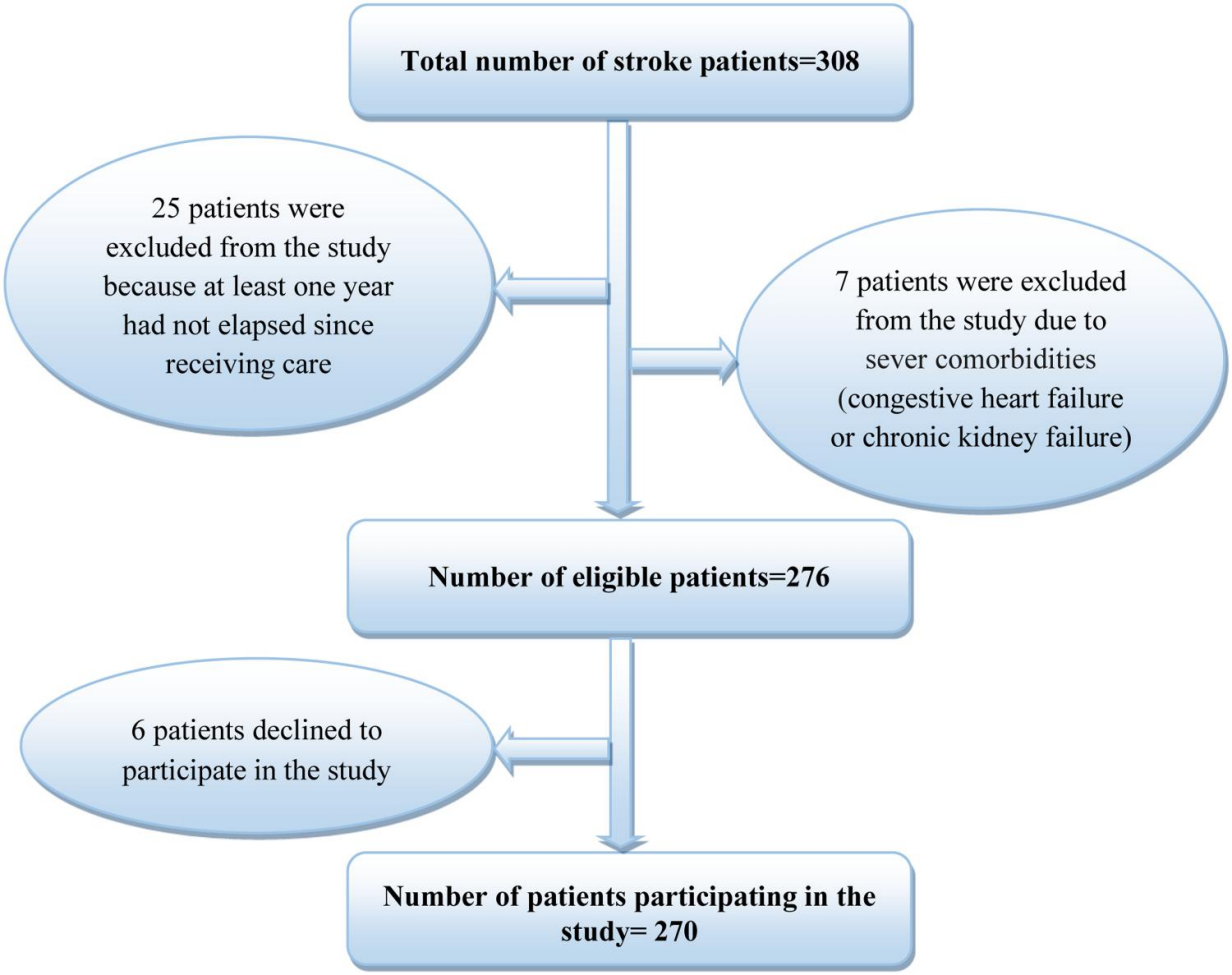
Flow figure of patients participating in the study.

### 2.2 Study Tool

The data collection tool was a researcher‐designed questionnaire. The initial draft was developed by reviewing similar studies, examining cost components based on care records, and interviewing patients and relevant physicians. Experts then confirmed the questionnaire's content validity, achieving scores of 0.96 and 0.94 for the CVR (Content Validity Ratio) and CVI (Content Validity Index), respectively. Moreover, the questionnaire's reliability was assessed using internal consistency and was approved, with a Cronbach's alpha of 0.883. The final questionnaire consisted of 58 questions: 20 addressed independent demographic and background variables related to the patients, 37 focused on cost dimensions of stroke, and 1 question covered patients' perspectives on the costs imposed on them [18].

### 2.3 Perspective and Time Horizon

This study was conducted from the patient/family perspective. We collected data in May and June 2024 and calculated and reported costs imposed on patients in the 1 year before data collection.

### 2.4 Cost Categories and Measurement

We determine the economic burden of disease by calculating the total cost imposed on patients and their families. Total cost includes direct medical, direct non‐medical, and indirect costs. Direct medical costs refer to all costs imposed on the patient and their family at the time of care, paid to the centers or individuals providing care. Direct non‐medical costs include items such as travel to care centers, making the work and home environment suitable and safe, providing support equipment, and expenses paid for personal matters. Indirect costs also refer to the opportunity cost for the patient and their family members, calculated as reduced income due to job loss or absence from work.

### 2.5 Data Collection and Analysis

This study was conducted from a patient/household perspective. The study collected costs incurred by patients and their households through interviews with patients and household members, and recorded responses in the corresponding questionnaire. Moreover, when possible, we used patients' records from service centers and clinical information systems to verify their responses, such as when calculating incurred direct costs. We used the human capital approach to estimate indirect costs by calculating and reporting the income lost by the patient (and their household members) due to exposure to the disease.

### 2.6 Valuation

Healthcare costs are based on the Central Bank of Iran exchange rate, expressed in USD ($1 = 370,777 IRR on June 4, 2024).

### 2.7 Statistical Analysis

We first analyzed the data descriptively and then statistically. In the descriptive part, we reported quantitative variables as mean (standard deviation) and qualitative variables as frequency (percentage). We used *t*‐tests and ANOVA to explore statistical relationships between demographic and background variables and patient costs, depending on whether the variables were dichotomous or had multiple categories. We also used the Tukey post‐hoc test to evaluate the significance of relationships between defined groups. All statistical analyses were conducted using SPSS version 27, with a significance level of *p* < 0.05.

### 2.8 Ethical Considerations

All patients were free to accept or decline participation and could withdraw at any stage. The research team collected data at appropriate times and in coordination with patients and their companions, ensuring no disruption to patient care. The researchers obtained written informed consent from the patients, assuring them that the study results would be used solely for their intended purposes and emphasizing the confidentiality and anonymity of their information. The Ethics Committee of Semnan University of Medical Sciences also approved the study (IR.SEMUMS.REC.1400.210).

## 3 Results

The mean age of the patients studied was 65.05 years (±13.04); approximately 13% were under 50, 17% were between 50 and 59, and over 70% were elderly. The gender distribution of disease prevalence was nearly equal between males and females, with a slightly higher rate in men. Two‐thirds of the patients were married. Less than one‐quarter (23%) had a university education, while about one‐third were illiterate. Retired individuals (41%) and homemakers (32%) were the largest occupational groups. Over three‐quarters (78%) of the patients lived in urban areas, with more than half (56%) residing in the provincial capital. Most (98%) had basic health insurance, 75% covered by Social Security, and more than two‐thirds (71%) had supplementary coverage. Ischemic strokes accounted for more than half (51%) of cases, followed by strokes of unknown origin and hemorrhagic strokes, which made up 38% and 10%, respectively. Nearly half (47%) of the patients sought follow‐up and definitive diagnosis immediately after symptoms appeared. This means that more than half of the patients, despite noticing early and unusual physical or mental symptoms of an illness, did not take seriously the need to consult a physician for examination and diagnosis. Nevertheless, the majority of them (98.1%) took steps to undergo the necessary follow‐ups and receive the required care after being diagnosed with the disease. Over half (51%) of the patients remained on continuous treatment, whereas more than a quarter (26%) received intermittent therapy from their physicians. Neurologists managed treatment in 95% of cases, and all patients received necessary care from public and private centers. The most common stroke complications observed were limb paralysis (26%), impaired consciousness (23.3%), and speech disorders (23%) [18] (Table 1). Also, income analysis indicated that the annual income of patients and their households was 192,751,000 Iranian Rials, equivalent to approximately $5198 [18].

**Table 1:** Demographic and background characteristics of patients.

Demographic characteristics	Grouping	Frequency	Percentage
Age	Less than 40 years	13	4.8
	40–49 years	21	7.8
	50–59 years	46	17
	60–69 years	79	29.3
	70–79 years	81	30
	80 years and older	30	11.1
Gender	Female	124	45.9
	Male	146	54.1
Marital status	Single	10	3.7
	Married	179	66.3
	Single/other	5	1.9
	Widowed	76	28.1
Educational level	Illiterate	91	33.7
	High school	66	24.4
	Diploma	50	18.5
	Associate's and bachelor's	52	19.3
	Masters	11	4.1
Employment status	Employee	26	9.6
	Manual worker	6	2.2
	Self‐employed	25	9.3
	Retired	112	41.5
	Homemaker	87	32.2
	Unemployed	14	5.2
Urban/rural	Urban	212	78.5
	Rural	58	21.5
Residing in the provincial center	Yes	151	55.9
Basic insurance	Yes	264	97.8
Insurance type	Social security	199	75.4
	Health service	49	18.6
	Other types of insurance	16	6.1
Supplemental insurance	Yes	192	71.1
Type of stroke	Ischemic	139	51.5
	Hemorrhagic	27	10
	Unknown	104	38.5
Time elapsed from the onset of symptoms to diagnosis	Immediately	128	47.4
	Less than 1 year	125	46.3
	1–4 years	17	6.3
Time elapsed from diagnosis to the start of care	Immediately	264	98.1
	Less than 1 year	3	1.1
	1–4 years	2	0.7
Monitoring status	Continuous	137	50.7
	Intermittent	71	26.3
	On demand	42	15.6
	Not being monitored	20	7.4
Treating physician	Neurologist	254	94.1
	Neurosurgeon	16	5.9
Place of care	A combination of private and public centers	270	100
Stroke complications	Facial paralysis	35	13
	Limb paralysis	70	25.9
	Speech disorders	62	23
	Impaired consciousness	63	23.3
	Others (numbness of hands and feet, bleeding eyes, etc.)	147	54.4

The analysis of costs incurred by stroke patients and their families shows they face annual expenses of 561,323,000 IRR ($1514), and the total economic burden of stroke on patients and their households in Semnan is estimated at approximately 151,557,260,000 IRR ($408,755.8). Of this, roughly 339,582,000 IRR (about $916) are direct medical costs, while around 108,370,000 IRR ($292) are direct non‐medical costs. Indirect costs total approximately 113,370,000 IRR ($306), representing 60.49%, 19.31%, and 20.20% of total expenses, respectively. Of direct medical costs, 5% is allocated to diagnostic services, 12% to physician and other therapist visits, and 83% to therapeutic care, primarily for necessary medications, hospital stays, and rehabilitation services. Of the other costs borne by patients, 108,370,000 IRR is classified as direct non‐medical costs, with payments to others (for personal activities and daily life) making up 47% and supportive equipment (wheelchairs, walkers, etc.) accounting for 31%. Additionally, an amount of 113,370,000 IRR is considered as indirect or opportunity costs (due to income loss caused by the disease) for patients and their families, of which 79,960,000 IRR (70%) results from the patient's income loss, and 33,400,000 IRR (30%) from the income reduction of other household members (Table 2).

**Table 2:** The incurred economic burden on patients with stroke and their households.

Categories of incurred costs	Types of incurred costs	IRR mean (std. dev.)	USD mean (std. dev.)
Direct medical costs	Laboratory	8,201,900 (±13,301,310)	22.12 (±35.87)
	Sonography	1,759,600 (±5,675,950)	4.74 (±15.31)
	MRI	5,537,800 (±12,431,940)	14.93 (±33.53)
	Other diagnostic services	1,808,600 (±28,682,100)	4.88 (±77.35)
	Visit by doctors	39,222,593 (±47,280,298)	105.78 (±127.51)
	Surgery	8,670,400 (±49,740,730)	23.38 (±134.15)
	Hospitalization	54,550,200 (±125,002,160)	147.12 (±337.13)
	Medication and supplements	139,940,700 (±138,259,650)	377.42 (±372.89)
	Home care	20,000,000 (±75,535,070)	53.94 (±203.72)
	Rehabilitation services	59,890,800 (±130,385,640)	161.53 (±351.65)
	Total	339,582,000 (±290,556,278)	915.86 (±783.64)
Direct non‐medical costs	Paying others for implementing daily affairs	51,088,900 (±199,362,510)	137.79 (±537.67)
	Purchase of supportive devices	34,133,300 (±59,351,550)	92.06 (±160.07)
	Adjusting the environment	11,240,700 (±55,366,620)	30.31 (±149.32)
	Travel to care settings	11,907,800 (±32,300,040)	32.11 (±78.11)
	Total	108,370,000 (±245,937,633)	292.27 (±663.30)
Indirect costs	Loss of a patient's job	51,814,800 (±257,679,170)	139.74 (±964.97)
	Loss of the patient's job position	2,963,000 (±38,447,070)	7.99 (±103.69)
	Decrease in patient income due to absence from work	25,185,200 (±142,395,080)	67.92 (±384.04)
	Loss of a job for other household members	21,111,100 (±166,271,900)	56.93 (±448.44)
	Loss of a job position for other household members	0	0
	A decrease in household members' income due to absence from work	12,296,300 (±114,741,930)	33.16 (±309.46)
	Total	113,370,000 (±346,415,704)	305.76 (±934.29)
Total costs		561,323,185 (±616,607,509)	1513.91 (±1663.01)

The review of stroke prevalence in Iran shows that the disease affects 150 people per 100,000, which is about 50% higher than the global average. Additionally, the average age at which stroke occurs in Iran is around 65–70 (65 in the study conducted in Semnan Province), which is roughly 10 years younger than the global average of about 75–80. Considering Iran's population of 86 million in the year of the study (1403 in the Iranian calendar and 2024 AD), the estimated number of stroke cases is 129,000. Studies indicate that there is no published research in Iran regarding the financial burden of stroke on patients, nor is there any relevant data available from health authorities such as the Ministry of Health. Furthermore, while calculating the national burden of stroke is crucial for health policymaking, conducting similar studies across regions would be prohibitively expensive and time‐consuming; therefore, cautiously extrapolating this study's results to the national level can provide insight into the situation. Given the average cost per stroke patient and their household is 561,323,185 IRR ($1513.91), it can be estimated that the economic burden of this disease on patients and their households in the year 2024 (assuming that the situation and burden on patients are similar on a national scale) is approximately 72 trillion, 410 billion, 690 million, and 865 thousand IRR ($195 million, $294 thousand, and $390).

The statistical analysis of the relationship between demographic and background variables and the total costs imposed on patients and their households shows that, in the initial analyses, only the variables of marital status, occupational status, patient follow‐up status, household income, having supplementary health insurance, and the patient's experience of stroke complications had a statistically significant association with the total costs imposed on them. In subsequent analyses using follow‐up tests, the statistically significant association of all these variables—except for the patient follow‐up status—was confirmed. The results indicate that costs for patients who have lost their spouses are higher than those for married patients; costs are higher for workers compared to employees and retirees; patients with lower income face higher costs than those with higher income; patients without supplementary insurance incur higher costs than those with it; and patients experiencing severe stroke complications (such as paralysis of the face and limbs or speech and cognitive impairments) face higher costs than those without such complications (Table 3).

**Table 3:** Statistical relationship of demographic/background variables with the incurred economic burden of patients with stroke and their households.

Demographic/background variables		Basic group (I)	Compare group(s) (J)	Mean difference (I−J)	Std. error	Sig.	95% Confidence interval	
							Lower bound	Upper bound
Marital status		Widowed	Single	363,877,631 (981.39)	203,248,849 (548.17)	0.280	−161,590,687 (−435.81)	889,345,950 (2398.6)
			Married	288,755,788^a^ (778.78)	82,722,270 (223.1)	0.003	74,890,212 (201.98)	502,621,364 (1355.58)
			Divorced	518,027,631 (1397.14)	278,956,431 (752.35)	0.249	−203,170,875 (−547.95)	1,239,226,138 (3342.24)
Employment status		Manual worker	Employee	946,851,795^a^ (2553.69)	272,837,026 (735.85)	0.008	163,598,508 (441.23)	1,730,105,082 (4666.16)
			Self‐employed	573,579,333 (1546.96)	273,858,253 (738.6)	0.293	−212,605,667 (−573.4)	1,359,764,333 (3667.33)
			Retired	735,323,333^a^ (1983.19)	252,433,497 (680.82)	0.044	10,643,955 (28.7)	1,460,002,712 (3937.68)
			Homemaker	656,938,046 (1771.78)	254,271,005 (685.77)	0.105	−73,016,401 (−196.92)	1,386,892,493 (3740.5)
			Unemployed	390,527,619 (1053.26)	293,944,928 (792.78)	0.769	−453,321,674 (−1,222.62)	1,234,376,912 (3329.16)
Monitoring status		Continuous	Intermittent	105,128,058 (283.53)	89,270,234 (240.76)	0.642	−125,666,262 (−338.92)	335,922,378 (905.99)
			On demand	261,865,704 (706.26)	107,672,811 (290.39)	0.074	−16,505,620 (−44.51)	540,237,027 (1457.04)
			Not being monitored	287,147,846 (774.44)	146,129,874 (394.11)	0.204	−90,648,238 (−244.48)	664,943,931 (1793.37)
Annual income of households		< 50 Million IRR	50–100 Million IRR	576,506,111 (1554.85)	268,541,592 (724.26)	0.204	−161,084,366 (−434.45)	1,314,096,588 (3544.16)
			100–150 Million IRR	692,560,268^a^ (1867.86)	212,800,106 (785.88)	0.011	108,072,294 (291.47)	1,277,048,243 (4716.23)
			150–200 Million IRR	598,618,412^a^ (1614.49)	217,014,378 (585.29)	0.048	2,555,295 (6.89)	1,194,681,531 (3222.1)
			> 200 Million IRR	625,896,023^a^ (1688.06)	212,396,523 (572.84)	0.029	42,516,551 (114.66)	1,209,275,496 (3261.46)
Supplemental insurance		Yes	No	−243,342,395 (−656.3)	94,003,236 (253.53)	0.016	−442,623,356 (−1193.77)	−51,494,608 (−138.88)
Stroke complications	Facial paralysis	Yes	No	270,283,902 (728.96)	105,030,296 (283.27)	0.013	73,501,029 (198.23)	477,548,275 (1287.96)
	Limb paralysis	Yes	No	824,336,557 (2223.26)	93,751,636 (252.85)	0.001	646,485,141 (1,743.59)	1,011,215,789 (2727.28)
	Speech disorders	Yes	No	265,411,979 (715.82)	94,731,554 (255.49)	0.007	82,223,123 (221.75)	448,847,905 (1210.56)
	Impaired consciousness	Yes	No	248,978,040 (671.5)	91,767,452 (247.5)	0.008	67,371,084 (181.7)	439,111,961 (1184.3)
	Mild complications	Yes	No	153,243,282 (413.3)	76,973,138 (207.59)	0.054	−5,078,585 (−13.69)	305,724,920 (824.55)

a The mean difference is significant at the 0.05 level. The review of patients' perceptions of the costs they face due to the disease shows that, out of 270 surveyed patients, 23 (8.51%) considered the costs negligible, 39 (14.44%) found them bearable, 61 (22.60%) viewed them as heavy, and 147 (54.45%) regarded them as unbearable (Figure 2).

**Figure 2:**
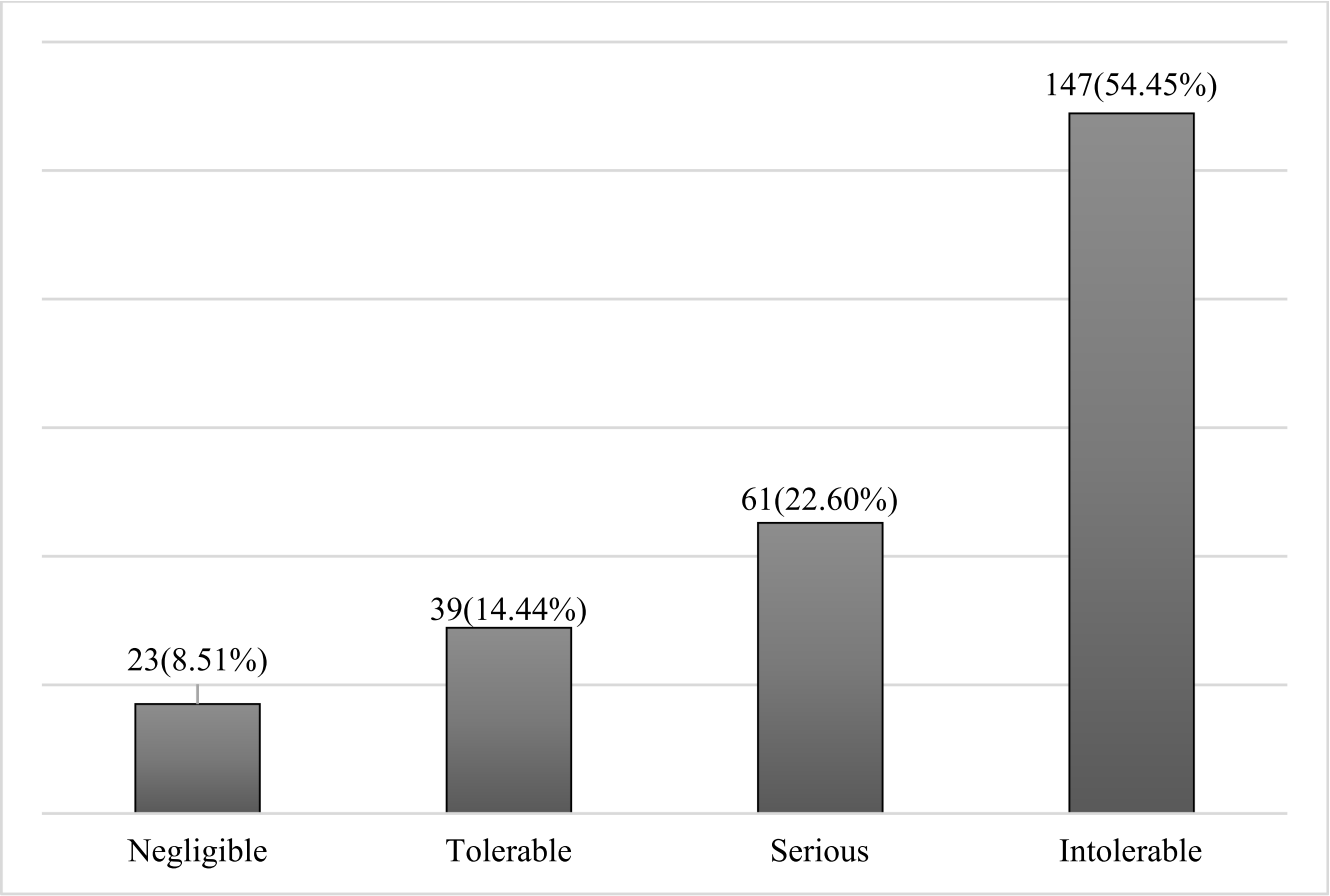
The patient's perception of incurred costs due to stroke in the last 12 months.

## 4 Discussion

The findings of this study show that, on average, an annual cost of 561 million and 323 thousand IRR ($1514) is imposed on patients with stroke and their households. Of the total expenses incurred by patients and their families, approximately 60% is attributed to direct medical costs, totaling 339 million and 582 thousand IRR ($916). Direct non‐medical and indirect costs each account for around 20%, corresponding to approximately 108 million and 370 thousand rials ($292) and 113 million and 370 thousand rials ($306), respectively. A 2017 study conducted in Sweden estimated that the average costs imposed on stroke patients were $37,000 and $50,000 in the first and second years after the event, respectively [19]. A 2016 study in Lebanon showed that the daily cost of hospitalization and care due to stroke was about $538, with an average hospitalization cost per patient during the treatment period of around $6961. This study also reported annual hospital costs per stroke patient of approximately $7130 in high‐income countries, $9688 in the United States, $1917 in Turkey, and $1578 in Pakistan [16]. Another similar study conducted between 2006 and 2013 in Guangzhou, China, estimated the hospitalization cost for stroke patients at $3212; in contrast, this cost was $20,396 in the United States in 2008, $6845 in the Netherlands in 2012, $8104 in Sweden in 2001, $10,190 in Singapore in 2012, $8732 in South Korea in 2007, and $8662 in Japan in 2002 [20]. Another study estimated healthcare costs related to stroke per patient to be nearly equal between the United States (€3875) and Europe (€3483) [14].

A study conducted in European countries estimated the cost of lost economic productivity due to stroke (indirect costs) at approximately €12 billion, attributing this to premature death and work absenteeism among patients [14]. It also reported that about 3.1 billion hours of informal care were provided to these individuals, with an estimated cost of around €16 billion [14]. Another study assessed the indirect costs imposed on stroke patients in Germany in 2012 at approximately $2960 per patient [10]. This study further showed that the lifetime indirect costs after stroke amount to $22,243 for men and $11,765 for women in Sweden [10]. Additionally, it highlighted that the share of indirect costs in the total costs caused by stroke varies widely—from 3% in Australia to 71% in the United States and Spain—but typically, indirect costs account for about one‐third of the total costs [10].

Given the national prevalence of stroke and the average cost imposed by the disease, the economic burden on patients and their households in the year 1403 (2024) is estimated at approximately 72 trillion and 410 billion IRR (equivalent to $195 million and 294 thousand). A similar study conducted in 2017 across 32 European countries found that stroke cost about 60 billion euros that year, with 45% of that amount—approximately €27 billion—being healthcare‐related expenses. This figure accounts for 1.7% of the total healthcare expenditures in those countries. This also amounts to €59 per European citizen, ranging from €11 in Bulgaria to €140 in Finland [14]. In the United States, the annual cost of stroke‐related care is estimated at around $18.8 billion [21], while in China, it is estimated to be about $6 billion [22].

An analysis of the statistical relationship between demographic and background variables and the total costs incurred by patients and their families shows that costs are higher for patients whose spouses have died compared to married patients, higher for workers compared to employees and retirees, higher for low‐income patients compared to those with high income, higher for patients without supplementary insurance compared to those with it, and higher for patients experiencing severe stroke complications compared to others. The increased costs for patients with widowed spouses can be linked to decreased social and especially insurance support, as well as their generally older age. Similarly, the higher costs for workers and low‐income groups may be due to their limited financial ability to undergo regular check‐ups and timely periodic care, or their lack of access to comprehensive insurance systems, particularly supplementary insurance, which leads to more severe strokes and complications. Additionally, the higher treatment costs for patients without supplementary insurance can be attributed to the significant role of supplementary health insurance in reducing high healthcare expenses. The need for ongoing care and the complexity and high costs of the necessary medical services explain the increased costs for patients with severe disease complications.

A 2017 study conducted in Sweden identified the type and severity of stroke‐related complications as the main factors influencing the burden of disease on stroke patients, with the annual costs ranging from €10,000 to €120,000, depending on these factors [19]. A 2015 study in Denmark found that stroke type was the primary determinant of costs, estimating direct medical costs at €10,720, €8205, and €7377 for hemorrhagic, ischemic, and undetermined origin, respectively. The average costs for patients in the first 5 years after stroke onset were €2771, €2515, and €2432 for these three stroke types, respectively [11]. Additionally, annual indirect costs due to the income loss of the patient's spouse, who provides care, were estimated at €989, €1544, and €1645 for these same stroke types [11]. Similar studies have shown that factors such as age, gender, type of treatment, comorbidities like congestive heart disease and diabetes, and stroke type influence hospitalization costs [23]. A study conducted in Lebanon highlighted the type of stroke as an essential factor in patient costs, identifying hemorrhagic stroke as the most expensive and transient ischemic attack as the least costly [16]. This study also pointed to hospital stay duration, length of stay in the intensive care unit, stroke type and severity, insurance or payer coverage, and infectious complications as key factors affecting direct medical costs [16]. Research in China indicated that stroke type, insurance type, age, comorbidities, disease severity, length of hospitalization, and hospital level significantly influence inpatient costs [20]. Another Chinese study reported out‐of‐pocket expenses of $484.8 per stroke patient in underprivileged rural areas in 2008 [24]. This study also emphasized the importance of intervention improvements, showing that the incidence of catastrophic health expenditures among stroke patients was 40.9%, which decreased to 30.9% after insurance reform [24].

### 4.1 Practical Recommendations

Based on the study results, these practical recommendations can be provided: educating the public about the importance of strokes and prevention methods, especially from a young age, through experienced healthcare personnel; creating and implementing a stroke care package within primary healthcare services, highlighting the need for timely screening and treatment; stresses efforts to improve general literacy and public health awareness; expand health insurance coverage based on universal health coverage principles, and establish suitable incentives for healthcare providers—particularly in primary prevention—to empower communities for self‐care; expand high‐quality treatment and rehabilitation services for stroke patients in the public sector; government support for domestic pharmaceutical and medical equipment manufacturers to achieve self‐sufficiency; focusing enhancement efforts on vulnerable groups facing catastrophic healthcare costs, mainly low‐income individuals and those with severe complications from the disease, to improve the current situation. This proposed package closely aligns with interventions suggested in other studies, which also emphasize identifying at‐risk individuals for appropriate care [25], efforts to identify and remove or mitigate disease risk factors [13], developing targeted public health policies focused on prevention [16], designing and implementing insurance interventions to improve conditions [24], and paying attention to targeted payment mechanisms to enhance care and reduce healthcare costs [26].

### 4.2 Study Strengths and Limitations

The study's strengths include its comprehensive approach to covering all aspects of costs and identifying relevant determinants. Its limitations include the inability to include deceased patients and the lack of responses from a small number of patients due to their extremely severe health conditions, as including these individuals could have led to a more accurate (and likely higher) estimate of actual costs. Another limitation is the exclusion of expenses related to basic and supplementary health insurance premiums for patients and their households, which were not considered in any of the cost categories examined, despite these expenses potentially significantly increasing overall healthcare costs. Although the research team attempted to reduce recall bias by interviewing patients' informed family members and reviewing information in patients' care records and clinical information systems, the possibility of this bias cannot be ruled out, given the patients' physical conditions and the 1‐year time frame considered for examining costs. Additionally, this economic evaluation was conducted from the perspective of patients and their families and excluded many other fees imposed on the healthcare system and society due to the disease; exploring these will require future studies. Another notable limitation is the calculation of the national economic burden of stroke on patients, which assumed that cost patterns in Semnan are similar to those in other parts of Iran; therefore, interpret the findings in this section with caution. Although this study attempted to examine all relevant variables—and their impact on the financial burden of stroke borne by patients—within the framework of demographic and background factors, we cannot rule out the possibility that other variables may act as residual confounders. Although bivariate analyses were performed in this study, conducting precise assessments to eliminate the influence of confounding variables using logistic regression applies to binary dependent variables (such as the presence or absence of a disease) and is incompatible with the nature of this study. Given that the stroke type was classified as “unknown” in the clinical records of more than one‐third of the patients, precisely determining the type might have revealed a significant statistical association between the stroke type and the financial burden imposed on the patients. Furthermore, we excluded seven patients due to comorbidities; if we had obtained data on the costs they incurred, the calculated financial burden of the disease would have been higher.

## 5 Conclusion

The study results showed that most stroke patients face a broad and significant range of healthcare costs, with their ability to pay being limited due to low‐income levels in Iran and a severe reduction in their financial capacity and that of their families to engage in productive economic activities. Beyond identifying the amount and types of stroke‐related costs, this study also highlighted demographic and contextual factors that influence the severity of these costs for patients and their households. These findings can help healthcare managers and policymakers design targeted, effective interventions.

## Author Contributions

**Masood Bagheri:** conceptualization, methodology, software, investigation, writing – original draft, supervision. **Sayed Amir Mortazavi:** conceptualization, data curation, investigation, visualization, writing – original draft, formal analysis. **Farid Gharibi:** conceptualization, methodology, software, data curation, investigation, formal analysis, supervision, writing – review and editing. **Yegane Partovi:** conceptualization, methodology, investigation, formal analysis, writing – review and editing.

## Conflicts of Interest

The authors declare no conflicts of interest.

## Transparency Statement

Farid Gharibi affirms that this manuscript is an honest, accurate, and transparent account of the study being reported, that no important aspects of the study have been omitted, and that any discrepancies from the study as planned (and, if relevant, registered) have been explained.

## Data Availability

Data available on request due to privacy/ethical restrictions.
